# Approbation of body investment scale on youth sample in Azerbaijan

**DOI:** 10.1192/j.eurpsy.2021.2185

**Published:** 2021-08-13

**Authors:** A. Ryzhov, N. Abanina, E. Sokolova, L. Pechnikova, A. Korneev

**Affiliations:** 1 Faculty Of Psychology, Lomonosov MSU, Moscow, Russian Federation; 2 Baku Branch, Lomonosov MSU, Baku, Azerbaijan

**Keywords:** suicide assessment, body attitudes, BIS

## Abstract

**Introduction:**

Body attitudes may serve as both vulnerability and protective factors for various forms of emotional and behavioral disorders, including suicidal and self-harm behaviors in adolescent and youth populations. Body Investment Scale (BIS, Orbach & Mikulincer, 1998) is an instrument specially designed to account for body attitudes in suicide assessment.

**Objectives:**

The study was aimed to provide a preliminary evidence for using the BIS translation in the assessment of suicide risk factors in Russian-speaking student population in Azerbaijan.

**Methods:**

The common recommendations for test translation were used. The factor structure, inter-item consistency of scales, and retest reliability were assessed. The participants were 100 students (18-23 years, 40 females, 60 males), 50 of them completed the retest.

**Results:**

The exploratory factor analysis with fixed number of factors reveals a homologous structure to the original BIS scales, explaining 48.2% of variance (in comparison to 55% of original measure). Inter-item reliability coefficients were lower: .989 for Body attitude, .696 for comfort with touch, .65 for Care and .61 for Protection scales. Pearson’s r for retest reliability (in a month) were above 0.9. Three items that could be excluded for enhancing the consistency of scales address physical contact and self-harm issues and might be culturally inappropriate.

**Conclusions:**

BIS is a promising instrument due to its grounded factor structure, but refinement of some items of the Russian translation is desired, as well as further study of applicability for adolescent population. BIS could fill the gap in scarcity of instruments for suicide assessment for Russian speaking population.

**Disclosure:**

No significant relationships.

